# Economic and operational impact of an improved pathway using rapid molecular diagnostic testing for patients with influenza-like illness in a German emergency department

**DOI:** 10.1007/s10877-018-00243-2

**Published:** 2019-01-04

**Authors:** Matthias Brachmann, Katja Kikull, Clemens Kill, Susanne Betz

**Affiliations:** 1 bcmed GmbH, Neue Strasse 76, 89073 Ulm, Germany; 2 Witten/Herdecke University, 58448 Witten, Germany; 3 Ategris hospitals, CEO’s Office, 45468 Muelheim, Germany; 4 Center for Emergency Medicine, Essen University Hospital, 45147 Essen, Germany; 5 Department of Emergency Medicine, University Hospital Marburg, 35033 Marburg, Germany

**Keywords:** Economic analysis, Emergency department, Influenza-like illness, Molecular test, Process optimization, Rapid diagnostic test

## Abstract

To evaluate the economic and operational effects of implementing a shorted diagnostic pathway during influenza epidemics. This retrospective study used emergency department (ED) data from the 2014/2015 influenza season. Alere i influenza A & B rapid molecular diagnostic test (RDT) was compared with the polymerase chain reaction (PCR) pathway. Differences in room occupancy time in the ED and inpatient ward and cost differences were calculated for the 14-week influenza season. The process flow was more streamlined with the RDT pathway, and the necessary isolation time in the ED was 9 h lower than for PCR. The difference in the ED examination room occupancy time was 2.9 h per patient on a weekday and 4 h per patient on a weekend day, and the difference in the inpatient room occupancy time was 2 h per patient on a weekday and 3 h per patient on a weekend day. Extrapolated time differences across the influenza season were projected to be 2733 h in the ED examination room occupancy and 1440 h in inpatient room occupancy. In patients with a negative diagnosis, the RDT was also estimated to reduce the total diagnostic costs by 41.52 € per patient compared with PCR. The total cost difference was projected to be 31,892 € across a 14-week influenza season. The improved process and earlier diagnosis with the RDT pathway compared with conventional PCR resulted in considerable savings in ED, inpatient room occupancy time and cost across the influenza season.

## Introduction

Seasonal influenza epidemics are a huge global burden. They typically infect 5–15% of the population during an epidemic [1], cause 250,000–500,000 deaths annually worldwide [2], and are associated with a significant number of related hospitalizations [3]. A fast diagnosis enables infection control measures and patient treatment to be initiated in a timely manner and also helps to optimize the use of bed spaces [4].

The molecular techniques that are commonly used to diagnose influenza, such as reverse transcriptase polymerase chain reaction (PCR), require samples to be transported to a laboratory, where molecular testing may not be performed immediately. There is, therefore, a delay before the results are available, which is a particular issue during weekends and public holidays, when laboratory diagnostic facilities are frequently unavailable [5, 6], and during influenza epidemics, when diagnostic laboratories do not have the capacity to sustain demand [7]. This has implications for patient flow and infection control [8], and patients may need to remain in multi-bed rooms in the emergency department (ED) until a suitable room becomes available [9]. Furthermore, delays in the diagnosis of influenza have also been associated with inferior outcomes, including disease progression and mortality [8, 10].

Rapid diagnostic tests (RDTs) for influenza have previously been limited by the poor sensitivity of antigen detection tests [11]. Highly accurate molecular RDTs are now available and are likely to be of considerable use in the ED because this is where patients with influenza-like illness (ILI) most commonly present [12, 13]. The benefits of RDTs reported in previous studies include a reduction in the mean waiting time in the ED [14, 15] and the length of stay [14, 16, 17]. Rapid diagnostic testing for influenza would therefore be expected to ease capacity constraints, reduce the strain on EDs during epidemics, make it easier to comply with infection control requirements, and improve the patient experience. Rapid diagnostic testing also reduces the prescribing of antibiotics [16, 18, 19], which are often prescribed inappropriately to influenza patients, contributing to the development of antibiotic resistance [16, 18]. It also reduces the need for laboratory tests and chest X-rays, with a subsequent reduction in costs [17].

The Alere™ i Influenza A&B rapid test (Alere, Waltham, USA) was chosen for this study because it is an RDT that utilises the enzyme-mediated molecular amplification of nucleic acids to yield a qualitative diagnostic result within 15 min [20]. It does not require sample preparation and can be used near to the patient outside of the laboratory. Whilst the performance of this RDT has been established (94.8% positive percent agreement for Influenza A and 98.4% for Influenza B, 97.7% negative percent agreement for A and 99.4% for B measured against PCR [20, 21]), there is a paucity of data on the economic benefits of implementing an RDT into the diagnostic pathway in the ED during an influenza epidemic. This retrospective study was designed to evaluate the operational and economic benefits of a shortened diagnostic pathway using this RDT for managing suspected influenza cases in the ED of a hospital in Germany during an influenza epidemic.

## Methods

**Fig. 1:**
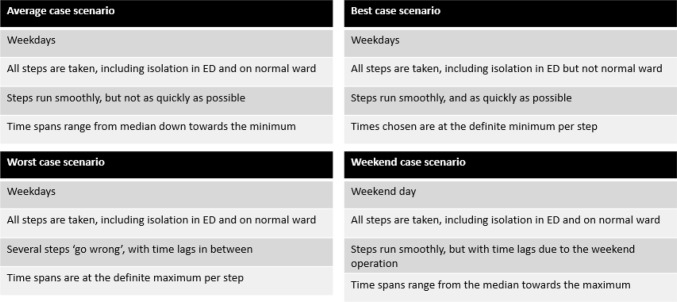
The conceptual model and its case scenarios (*ED* emergency department)

This retrospective analysis was based on data obtained from an ED that has approximately 56,000 emergency patients per year. The diagnostic pathways with PCR and the RDT in the management of patients with ILI, and their actual time requirements, were first mapped based on insight obtained from specialised hospital personnel (Head of ED, senior medical/nursing staff and a virologist). A conceptual model was created (Fig. 1) to enable process times to be compared between the two diagnostic pathways. The scenarios depicted represent those most commonly encountered in clinical practice and include a best, average and worst case scenario, each relating to a normal weekday. Additionally, a weekend average case was also constructed to reflect the diagnostic operation out of hours. The process times for the RDT and PCR pathways, the typical length of stay in the ED, and the time that ED and inpatient rooms were occupied or unusable pending decontamination was obtained for each possible case scenario from the ED and virology staff. Data on the number of patients and their respective test results were obtained from the RDT device.

The number of patients tested was obtained from ED data relating to one week of the influenza epidemic in early 2015 (9th–15th March), during which time the rapid test had been performed by staff in the ED in accordance with the manufacturer’s instructions. PCR, which included confirmatory testing for negative rapid test results, was performed by a virologist on the same sample at an onsite diagnostic laboratory for all patients (PCR-Kit 1.0 Real Star®, Altona Diagnostics, analytic sensitivity for Influenza A: 0.45 copies/µl Influenza B: 2.42 copies/µl, analytic specificity: no cross-amplification with other respiratory pathogens detectable [22]). The proportion of patients that were tested and the ratio of weekday to weekend tests were calculated by comparing test data from the RDT device with patient statistics from the ED. The time and cost effects of both pathways were calculated per patient for the average weekday case scenario and the weekend case scenario (see conceptual model in Fig. 1). The effects per patient were then extrapolated to estimate the total effect across the influenza season.

The cost calculations were based on the cost of resources used for the management of patients with ILI and included expenditure related to the inpatient admission of patients who later received a negative diagnosis following PCR. The mean personnel and diagnostic unit costs assigned to every step of the two pathways, including the provision of technical personnel and diagnostic reagents, and the cost of performing the test were based on average rates which were either obtained from the ED or researched (see Table 1 for more detail). Incidental costs, such as the cost of protective clothing and its disposal, were researched. Inpatient reimbursement was based on the German base rate of 3231.20 € that was obtained from the National Association of Statutory Health Insurance Funds in Germany [23].

**Table 1:** Costs and other economic assumptions

Position	Value
Personnel costs ED nurse p.a.^a^	60,000 €
Personnel costs consultant ED p.a.^b^	111,000 €
Personnel costs cleaning staff p.a.^a^	36,000 €
Personnel costs radiographer p.a.^a^	50,000 €
Personnel costs transport service p.a.^a^	24,000 €
Net annual working time in h^a^	1650
PCR testing costs^a^	21.42 €
PCR on demand testing costs^a^	42.84 €
Alere i testing costs^c^	27.00 €

^a^bcmed project data

^b^ https://gehaltsreporter.de/gehaelter-von-a-bis-z/151.html

^c^Study price

All calculations that were based on data from the RDT device were adjusted to take account of false positive and negative results based on a specificity of 98.09% [24]. This was the lower of the two specificity values for influenza A and B respectively [24]. For calculations in which it was necessary to take account of the average hospital utilisation rate, a value of 77.4% was used, which is widely accepted as a representative figure in Germany [25]. All calculations were performed using Microsoft Excel.

## Results

During the 14 weeks of the 2015 influenza epidemic, the ED treated 15,300 emergency patients overall. Data obtained from the RDT for the analysed week of the 2015 influenza epidemic indicated that 5.3% of patients were tested for influenza; therefore, approximately 812 patients would have been tested during the whole influenza epidemic. 31% of the tested patients were positive. The positivity rate underlines the necessity for the preventive but costly isolation. The percentage ratio of patients tested on a weekday to patients tested on a weekend day was considered to be 64:36, based on these data.

### Process flow differences for the PCR pathway versus the RDT pathway

**Fig. 2:**
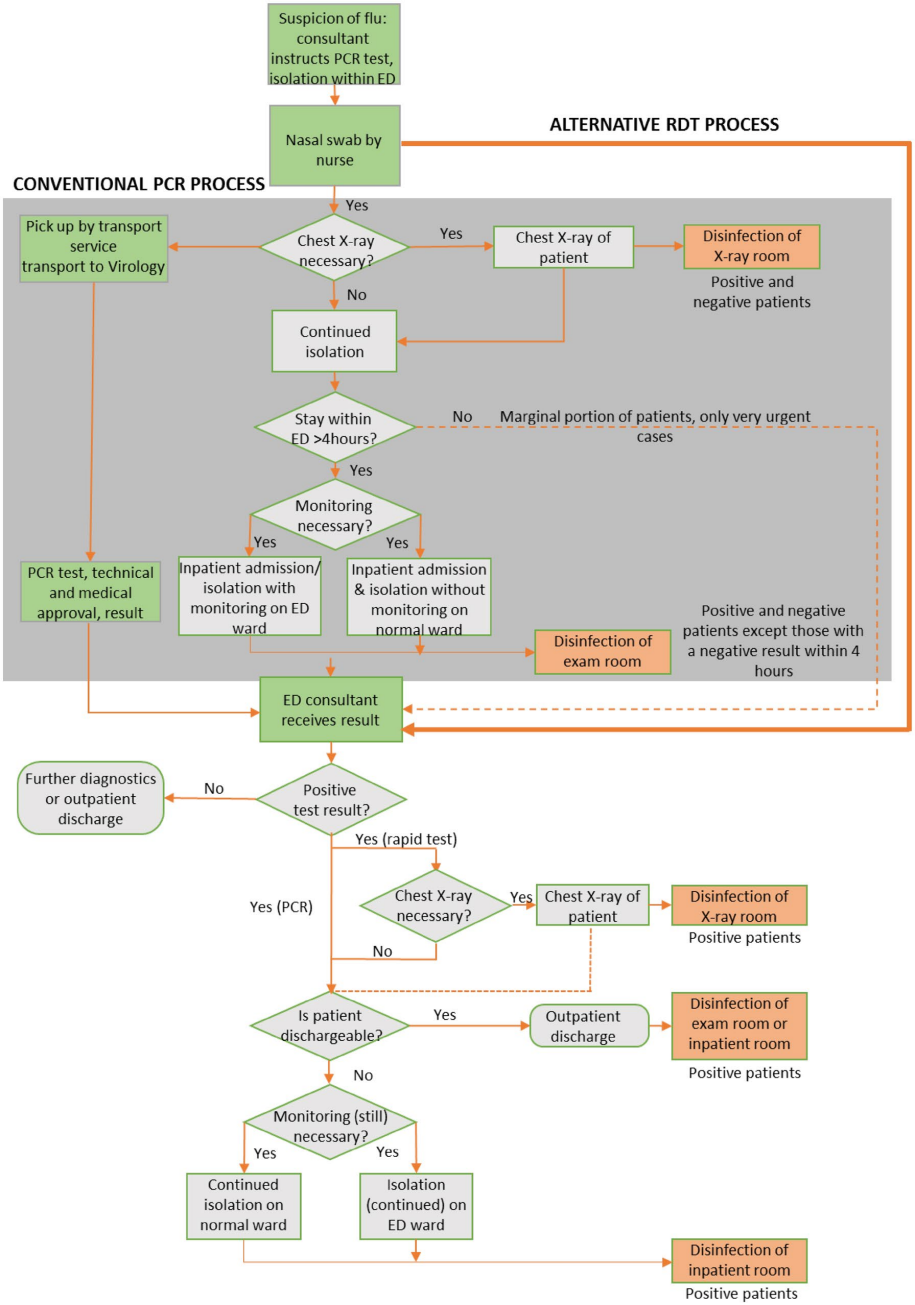
Comparison of the diagnostic process flow for PCR and the RDT (*ED* emergency department, *PCR* polymerase chain reaction, *RDT* rapid diagnostic test)

A conceptual flow diagram of the diagnostic process for influenza using either PCR or the RDT in the ED is presented in Fig. 2. This figure shows that the conventional pathway has more stages than the RDT pathway and also requires samples to be transported to a diagnostic laboratory. The uncertain diagnostic status of the patient prolongs the time during which patients must remain isolated in the ED and mandates the disinfection of multiple rooms (shown in orange in Fig. 2), including the X-ray suite and the ED examination room after inpatient admission. This is expensive and renders the room unusable, typically for 30 min. Patients diagnosed via this pathway often remain under isolation in the ED for over 4 h.

In the RDT pathway, a diagnosis is obtained directly from a nasal swab or nasopharyngeal swab in viral transport medium, and a result is typically available within 15 min. The continued isolation of patients and subsequent disinfection of the ED examination room and X-ray suite is only necessary when the rapid test returns a positive result. Patients who receive a negative result do not need to be kept under isolation, and hence spend less time in the ED examination room. Less time is also spent making provision for further isolation or inpatient bed space in the RDT pathway compared with the PCR pathway, which further improves patient flow and increases examination room availability in the ED. Discontinuing the isolation for negative results was possible since the described pathway was applied only in adult care and not the pediatric section with high prevalence of RSV and other highly contagious respiratory pathogens. The examination rooms were nonetheless cleaned with additional basis hygiene (wipe disinfection) but without the full disinfection program (residence time 30 min).

### Influence of rapid diagnostic testing versus PCR on the ED resource management and inpatient room occupancy

**Fig. 3:**
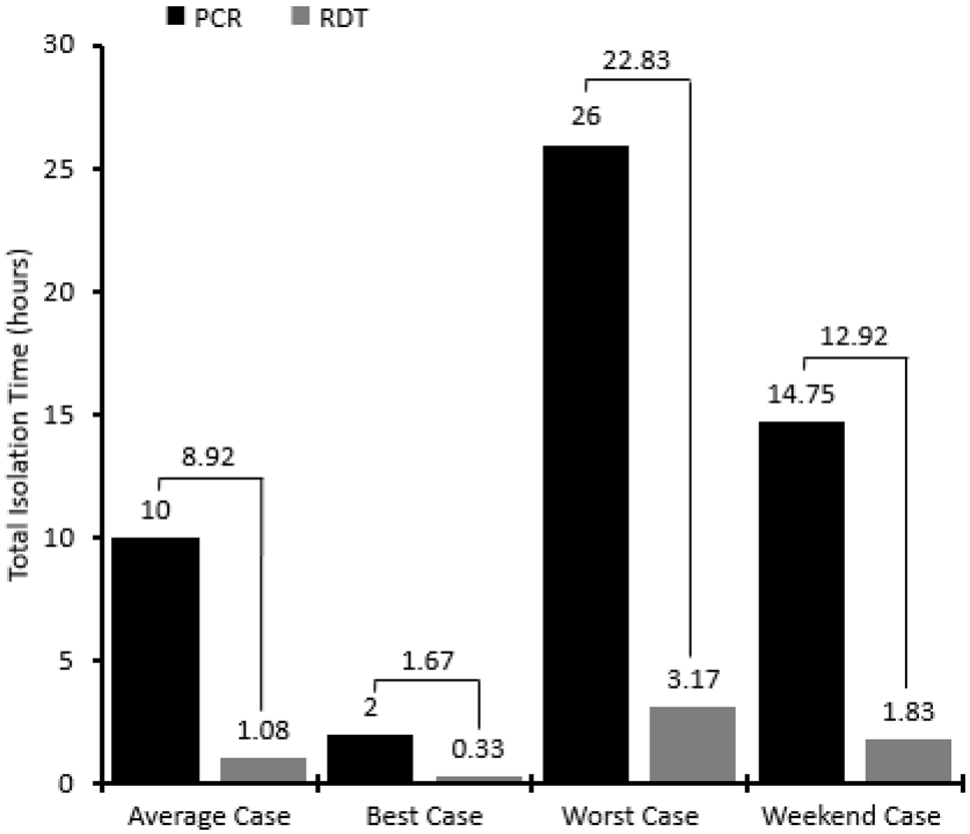
Total patient isolation times (*PCR* polymerase chain reaction, *RDT* rapiddiagnostic test)

Use of the RDT pathway in the ED was associated with a lower necessary isolation time (defined as the time elapsed between a medical practitioner considering flu as a potential diagnosis and confirmatory test results actually being available) in the ED for all scenarios depicted in the discrete model (Fig. 3). The necessary isolation time for the average case scenario was 10 h for the PCR pathway and 65 min for the rapid diagnostic pathway, representing a difference of almost 9 h. The difference was greatest for the weekend case scenario (Fig. 3).

The ED examination room occupancy time was shorter with the RDT pathway than the PCR pathway, with a difference of 2.9 h per patient on a weekday and 4 h per patient on a weekend day. Once extrapolated, this indicates a time saving of 2733 h across the 14-week influenza season (Table 2), which is the equivalent of 28 h of ED examination room time per day; hence, the RDT pathway could be expected to extricate a whole extra examination room in the ED per day. Similarly, the inpatient room occupancy was also lower with the RDT pathway, and there was a difference of 2 h per patient on a weekday and 4 h per patient on a weekend day, which is likely to be because the diagnosis is known in the ED so there is therefore less of a need for inpatient admission. This was extrapolated to indicate a reduction of 1440 h in inpatient room occupancy across the 14-week flu season (Table 2).

**Table 2:** Saved ED and inpatient room occupancy time

	Weekday (average) case	Weekend case	Flu season (14 weeks)
Patients tested for influenza (*n*)^a^	522	290	812^b^
ED examination room occupancy (h per case type)			
PCR pathway	4.5	7.5	
RDT pathway	1.58	3.33	
Difference	2.92	4.17	
Subtotal: Time saved (h) (patients*difference)	**1524**	**1209**	**2733**
Inpatient room occupancy (h per case type)^c^			
PCR pathway	2.08	3.28	
RDT pathway	0	0	
Difference	2.08	3.28	
Subtotal: Time saved (h) (patients*difference)	**1086**	**951**	**1440** ^d^

*ED* emergency department, *PCR* polymerase chain reaction, *RDT* rapid diagnostic test

^a^Assumes a weekday: weekend day ratio of 64:36

^b^Calculated as 5.3% of the number of emergency patients in one flu season (15,300)

^c^Inpatient ward and normal ward

^d^Excludes room capacity occupied as a result of false positives from the rapid test. Data from the rapid test device indicate that 31% of 812 patients (n = 252) received a positive rapid test result, so 560 patients would therefore have received a negative rapid test result. The specificity of the Alere™ i rapid test is 98.09% (manufacturers data), so the percentage of patients with a false positive result can be calculated as 1 − 0.981 × 560, equalling 10.7 patients. Assuming an average occupancy time is 3 days, and an average utilisation time of 77.4%, this would equal 596 h (10.7 pts × 3 days × 24 h × 0.774)

The average length of stay for inpatients in Germany is 7.4 days [22], which is the equivalent of 177.6 h. A total of 1440 h in saved ward capacity, which would therefore enable eight additional patients to be treated during the 14-week influenza season, with an anticipated reimbursement of 26,263 € (based on the German base rate for inpatient reimbursement of 3231.20 € per patient).

### Integrating rapid testing into the management of ILI Patients had significant economic benefits

The shortened diagnostic pathway with the RDT compared with conventional PCR was associated with less expenditure on caring for isolated patients in the ED, disinfecting the X-ray suite, and ED specialists searching for inpatient bed space. The number of times that protective clothing (protective gloves, protective gown, FFP3 mask, protective goggles) typically needs to be used was lower for the RDT pathway than the conventional PCR pathway. The calculated cost difference in patients who received a negative diagnosis with the RDT instead of PCR was €41.52 (Table 3). The costs for both pathways were totalled, and the combined cost difference for the average case and the weekend day scenario across a 14-week influenza epidemic was estimated to be 31,892 € (Table 4).

**Table 3:** Comparison of PCR and rapid test costs

Process stage	Key costs (€)	PCR pathway	RDT pathway	Difference
Diagnostic test	Cost of the test	21.42	27.00	5.58
	Cost of nursing staff to performing the rapid test		12.12	12.12
	Transport of samples to the lab (weekend)	0.61		− 0.61
Patient isolation in ED	Cost of labour to care for isolated patients	45.45	13.26	− 32.20
	Provision of protective clothing	2.25	0.30	− 1.95
	Disposal of contaminated waste	0.05	0.05	N/A
Possible X-ray^a^	Disinfection of X-ray unit/lost radiographer time	9.75	N/A	− 9.75
	Provision of protective clothing	0.25	N/A	− 0.25
	Disposal of contaminated waste	0.03	N/A	− 0.03
Inpatient admission^a^	Cost of ED specialists searching for bed space	13.05	N/A	− 13.25
Isolation on normal ward^a^	Lost revenue from unnecessary bed occupancy	N/A	N/A	
	Provision of protective clothing	1.37	N/A	− 1.37
	Disposal of contaminated waste	0.02	N/A	− 0.02
Total costs		94.25	52.73	41.52

*ED* emergency department, *PCR* polymerase chain reaction, *RDT* rapid diagnostic test

^a^Unnecessary costs for negative cases only

**Table 4:** Total costs for both pathways

	Weekday (average) case	Weekend case
Patients (n)	**522**	**290**
Total cost per patient (€)		
PCR pathway	82.97	105.52
RDT pathway	49.32	56.14
**Cost** **Difference**^a^	**33.65**	**49.38**
Total cost per season (€)^b^		
PCR pathway	43,307.79	30,621.25
RDT pathway^a^	25,744.68	16,292.72
**Cost** **Difference**^a^	**17,563.11**	**14,328.53**
**Total cost** **difference** **weekday and weekend(€)**	**31,891.64**	

*PCR* polymerase chain reaction, *RDT* rapid diagnostic test

^a^Subtotal of the PCR costs minus the RDT costs

^b^The cost of the rapid test itself and the associated personnel cost, which were calculated to be 2.40 € and 1.33 € in total for the average case and the weekend case respectively, were deducted

### Effects depending of the size of the ED

To estimate the cost and room occupancy reductions that could be expected with use of the RDT pathway instead of the PCR pathway for the detection of influenza in EDs of different sizes, the cost and room utilisation data were extrapolated to three representative examples of EDs with different patient numbers. The projected cost difference with the RDT instead of PCR during a typical flu season was estimated to range from 5633 € for a small ED with 10,000 patients annually to 33,649 € for a large ED with 60,000 patients annually (Table 5). Using the same extrapolation principles, the number of ED examination rooms extricated with use of the RDT instead of PCR was projected to range from 0.2 rooms per day for a representative small ED to 1.2 days per day for a representative large ED. The projected time difference in inpatient room occupancy on the ward enabled up to eight new inpatients to be admitted, with a projected remuneration of up to 30,000 € (Table 5).

**Table 5:** Extrapolation of differences in cost and room occupancy to ED departments of different sizes

	Small hospital	Medium hospital	Large hospital
Patient statistics (n)			
ED patients per year	10,000	30,000	60,000
Emergency patients tested for influenza per season^a^	143	428	856
Patients tested on weekdays during the flu season^b^	92	274	548
Patients tested on weekend days during the flu season	51	154	308
Patients testing positive during the flu season^c^	45	133	265
Patients testing negative during the flu season	99	295	591
Cost data (€)			
Difference per patient (cost of PCR − cost of RDT)^d^			
Average case	33.65	33.65	33.65
Weekend case	49.38	49.38	49.38
Difference per influenza season^e^			
Average case	3102	9220	18,440
Weekend case	2531	7605	15,209
Cost savings with the RDT per flu season	5633	16,825	33,649
ED examination room occupancy (h)			
Time difference per case (cost of PCR − cost of RDT)^f^			
Average case	2.92	2.92	2.92
Weekend case	4.17	4.17	4.17
Time difference per influenza season			
Average case	268.64	800.08	1600.16
Weekend case	212.67	642.18	1284.36
Total time difference	481.34	1442.26	2884.52
Hours per day^g^	4.91	14.72	29.43
Rooms per day^h^	0.21	0.61	1.2
Inpatient ward room occupancy (h)			
Time difference per case			
Average case	2.1	2.1	2.1
Weekend case	3.28	3.28	3.28
Time difference per influenza season			
Average case	193.2	575.4	1150.8
Weekend case	167.28	505.12	1010.24
Total time difference^i^	254.9	765	1529
Capacity for new inpatients (n)^j^	1.47	4.32	8.60
Expected revenue (€)^k^	4749.9	13,926.5	27,788.32

*ED* emergency department, *PCR* polymerase chain reaction, *RDT* rapid diagnostic test

^a^Assumes 5.3% of ED patients tested for influenza in a 14-week influenza season

^b^Assumes a weekday:weekend day ratio of 64:36

^c^Data from the RDT indicated that 31% of patients tested received a positive diagnosis for influenza, so this was calculated as 0.031 multiplied by the number of emergency patients tested for influenza per season, and the remainder were assumed negative

^d^See Table 4 for cost unit data

^e^Calculated as the difference per patient multiplied by the number of patients

^f^See Table 2 for cost unit data calculations

^g^Total time difference divided by 14 weeks multiplied by 7 days

^h^Hours per day divided by 24

^i^Excludes room capacity occupied as a result of false positives from the rapid test calculated as [(1 − 0.981)*n for negatives]*3 days*24 h*0.774 occupancy rate

^j^Calculated as available capacity/(average length of stay in hours) = available capacity/(7.4*24)

^k^Based on a remuneration rate of 3231.20 €

## Discussion

There is a paucity of evidence on the economic and functional benefits of using rapid molecular diagnostic tests for the detection of influenza during an influenza epidemic. This retrospective analysis used data obtained from an ED during an influenza epidemic to calculate differences in cost and room occupancy time with the simplified pathway using the molecular RDT compared with the conventional PCR pathway, and extrapolated the differences to estimate the actual savings across the 14-week flu season.

This study has demonstrated that patient flow in the ED is improved by implementation of a simplified patient pathway which used an RDT. Diagnosis in the ED with the rapid test eliminated the need for samples to be processed by a laboratory and reduced the time required to diagnose influenza, during which the patient must remain isolated, by 9 h in the average case.

Other similar studies investigating the benefits of early diagnosis have also reported that rapid diagnostic testing reduces the patient waiting time and isolation time in the ED [15, 16, 26] and a similar reduction in the ED waiting time of 9 h was reported in the prospective study of a rapid commercial PCR assay compared to an in-house PCR test by Soto et al. [15]. The most likely explanation is a reduction in the time spent awaiting a diagnosis. The same-day diagnosis with rapid testing enables infection control measures to be instigated in a timely manner [27], reducing the risk of nosocomial transmission, which prolongs hospital stays [17], warrants additional interventions and causes absenteeism among health workers [27]. Implementation of RDTs in the ED also enable an accurate diagnosis to be obtained in the ED [16]. Clinical diagnosis in the ED can otherwise be difficult because of the overcrowded environment and because ILI patients without complications are usually seen as less urgent when triaged [16]. Clinical diagnosis of influenza in the ED based on signs and symptoms alone has recently been reported to have sensitivity as low as 36% [28], leading to inappropriate isolation and treatment. In addition, rapid diagnostic testing in the ED and on the ward also reduces strain on diagnostic laboratories.

Previous studies have already established that reduced waiting times in EDs improve patient satisfaction [29]. A previous large-scale survey concluded that increased time spent waiting for provider care was inversely related to patient-reported experience scores, particularly a patient’s perceived confidence in the care provider and perceived quality of care [30]. It is therefore reasonable to postulate that the reduction in waiting time and improved patient flow with use of an RDT would improve patient satisfaction levels, although the evaluation of this was unfortunately outside the scope of the present study. Patient satisfaction scores are important because they are increasingly considered in reimbursement decisions in some countries [31] and influence the reputation of the ED [32]. The reduced time to diagnosis with the RDT may also reduce inpatient admissions by eliminating the need to admit patients with ILI symptoms who later receive a negative diagnosis, although this was also not evaluated explicitly in this study. However, the improved process flow with the RDT will help to overcome constraints in waiting room capacity, which is an issue during influenza epidemics and pandemics [33].

In this study, implementation of the RDT pathway was projected to reduce room capacity requirements per patient in the ED. Indeed, the estimated 2733 ED examination room hours saved over the course of an entire flu season is the equivalent to having a whole extra room in the ED free for each day of the epidemic, which reduces the pressure on ED resources. The availability of rooms is the limiting factor in many hospitals, and is a particular issue during the influenza season [34]. An increased availability of rooms because of shorter occupancy times will therefore enable more patients to be seen and reduce elopement rates, which also increase during influenza pandemics [34]. In hospitals where room capacity is not the limiting factor, the extra capacity can also be used for alternative clinical services, such as an additional examination room for an outpatient clinic. The increased patient turnover also has financial benefits, and the average ED patient in Germany has a reimbursement rate of 1675 € [35].

Implementation of the RDT pathway also reduced the time that patients who later receive a negative diagnosis need to remain isolated for prior to receiving their diagnosis. The estimated reduction in inpatient room occupancy time with use of the rapid test was 1440 h across the flu season. This is particularly advantageous because hospital beds are one of the scarcest resources during influenza epidemics, and the demand for bed spaces in general medicine wards for influenza-related cases can increase by over 50% during influenza epidemics, compared with 25–32% for other specialties [33]. A reduction in the unnecessary isolation time can enable more inpatients to be treated, and this retrospective analysis indicated that eight additional inpatients could receive treatment based on an average length of stay of 7.4 days [25].

This study also demonstrated the financial benefits of rapid diagnostic testing. By eliminating unnecessary costs with the PCR pathway in patients who later receive a negative diagnosis, implementation of the RDT pathway reduced the total per patient costs by 41.52 €. This was extrapolated to a combined cost saving for the average weekday case and weekend day case across the influenza epidemic of 31,892 €. Other studies have also reported that use of RDTs reduce the cost per patient [15], indicating that rapid diagnostic testing has a lower cost per patient than traditional laboratory-based molecular diagnostic tests.

The lower cost with rapid diagnostic testing is likely to be due to the reduction in labour time required to care for isolated patients in the ED and the elimination of subsequent treatment stages that are unnecessary in patients who receive a negative rapid test result. This eliminates the cost of disinfecting the X-ray suite and of ED personnel searching for bed space, and prevents loss of revenue from unnecessary bed occupancy on the inpatient ward. The disinfection of the X-ray suite also renders the room unusable for a considerable amount of time, during which radiographers are unable to work. Unfortunately, thorough disinfection of examination and X-ray suites is necessary while awaiting diagnostic results from PCR because influenza particles on non-porous surfaces can remain infectious for 24–48 h [27]. Protective clothing is also only worn for a single use because influenza virus infectivity can remain on the surface of protective clothing for 8 h or more [36], so the cost of protective clothing and its disposal was also taken into consideration in this study. By reducing the number of hours spent searching for bed space and awaiting the completion of the disinfection process for treatment areas, RDTs liberate staff time, which can be used to improve the care of ED patients.

The limitations of this study include the retrospective study design, which make it difficult to exclude all confounding variables, and the fact that data were extrapolated to different scenarios. However, allowances were made in the data analysis for confounding variables that were identified, such as the possibility of false positives with the rapid test. The data extrapolations were also based on real process data obtained from the University of Marburg ED and the RDT, which is unlikely to vary significantly over a single flu season. The RDT was performed in the ED of the University Hospital of Marburg, which is a typical ED, during the 2014 influenza epidemic. The findings are therefore likely to be relevant to other similar hospital EDs during an influenza epidemic. A further limitation is the influenza focus of this study. There are other respiratory diseases which also require patient isolation with admission to the hospital (e.g. RSV). Some patients will have to remain isolated even with a negative influenza test when their presenting complaints suggest other infectious respiratory causes. But there are also molecular RDTs for RSV available.

In summary, this comparative study demonstrates the benefits of implementing a molecular RDT into the diagnostic pathway in EDs during influenza epidemics for improving patient flow, reducing the requirement for ED and inpatient room space for influenza patients, and reducing the total costs of managing such patients. Further research comparing patient satisfaction among influenza patients who were diagnosed using ward-based rapid testing in comparison with patients diagnosed using laboratory-based molecular testing would also help to demonstrate the benefits of rapid diagnostic testing on the patient experience.
