# Correction to: Economic and operational impact of an improved pathway using rapid molecular diagnostic testing for patients with influenza-like illness in a German emergency department

**DOI:** 10.1007/s10877-019-00262-7

**Published:** 2019-02-14

**Authors:** Matthias Brachmann, Katja Kikull, Clemens Kill, Susanne Betz

**Affiliations:** 1 bcmed GmbH, Neue Strasse 76, 89073 Ulm, Germany; 2 Witten/Herdecke University, 58448 Witten, Germany; 3 Ategris Hospitals, CEO’s Office, 45468 Muelheim, Germany; 4 Center for Emergency Medicine, Essen University Hospital, 45147 Essen, Germany; 5 Department of Emergency Medicine, University Hospital Marburg, 35033 Marburg, Germany

## Correction to: Journal of Clinical Monitoring and Computing 10.1007/s10877-018-00243-2

The article Economic and operational impact of an improved pathway using rapid molecular diagnostic testing for patients with influenza-like illness in a German emergency department, written by Matthias Brachmann, Katja Kikull, Clemens Kill and Susanne Betz, was originally published electronically on the publisher’s internet portal (currently SpringerLink) on 04 January 2019 without open access.

With the author(s)’ decision to opt for Open Choice the copyright of the article changed on 30 January 2019 to © The Author(s) 2019 and the article is forthwith distributed under the terms of the Creative Commons Attribution 4.0 International License (http://creativecommons.org/licenses/by/4.0/), which permits use, duplication, adaptation, distribution and reproduction in any medium or format, as long as you give appropriate credit to the original author(s) and the source, provide a link to the Creative Commons license and indicate if changes were made. The original article has been corrected.

